# Cardiometabolic profile of 15057 elderly Spanish workers: association of sociodemographic variables and tobacco consumption

**DOI:** 10.1186/s12877-022-03547-w

**Published:** 2022-11-17

**Authors:** J. I. Ramírez-Manent, B. Altisench Jané, S. Arroyo Bote, C. López Roig, H. González San Miguel, A. A. López-González

**Affiliations:** 1 Investigation Group IUNICS, Palma, Spain; 2 Balearic Islands Health Service, Balearic Islands, Palma, Spain; 3 University of the Balearic Islands, Palma, Spain; 4 IDISBA, Balearic Islands Health Research Institute Foundation, Palma, Spain; 5 University School ADEMA Palma, Balearic Islands, Palma, Spain

**Keywords:** Heart disease risk factors, Sex, Smoking, Social class, Aging

## Abstract

**Background:**

Aging of the world population is one of the most significant demographic changes of our time. Populations older than 60 years are heterogeneous, and age is an independent cardiovascular risk factor aggravated by frailty, obesity, and diabetes, and influenced by several factors, including sex and socioeconomic status. The objective of this study was to calculate cardiovascular risk in workers of both sexes over 60 years of age and to assess whether there are difference s by sex, social class, smoking, and type of job.

**Methods:**

A cross-sectional study was carried out in 15,057 elderly Spanish workers from different autonomous communities in Spain and with different labor occupations. Anthropometric, sociodemographic, clinical, and laboratory values were determined. People were classified according to age from 60 to 64 years inclusive and from 65 to 69 years, smokers and non-smokers, and both blue-collar and white-collar workers. Subsequently, a multivariate analysis was carried out.

**Results:**

Men, blue-collar workers, smokers, and aging were factors that influenced cardiovascular risk: with an OR of 3.27 (95% CI: 2.64–4.05) in people 65 years of age or older versus the younger group, and an OR of 3.15 (95% CI: 2.69–3.69) in smokers versus non-smokers. A stronger independent association was found between smoking, age, and cardiovascular risk. The risk of developing non-alcoholic fatty liver and liver fibrosis was much higher in men than in women, with an OR of 4.06 (95% CI: 3.66–4.50) for the former and an OR of 2.10 (95% CI: 1.95–2.26) for the BARD index.

**Conclusions:**

The highest risk groups were observed in male subjects with a history of smoking and blue-collar workers and, as such, should be considered for cardiovascular risk screening programs.

## Introduction

Aging of the world population is one of the most important demographic changes of our time. The world population is aging rapidly: since 1980, the number of people aged 60 and over has doubled to approximately 810 million and is expected to grow to approximately 2 billion by 2050. Thus, it has been forecast that 22% of the total population will be over the age of 60 and about 5% will be over the age of 80 in 2050 [1].

Age is an independent cardiovascular risk factor in adults that is aggravated by other factors such as frailty, obesity, and diabetes [2–4]. The American Heart Association (AHA) found that the incidence of cardiovascular disease (CVD) in US men and women is approximately 75% between the ages of 60 and 79, and 86% in those older than 80 years [5]. In the same way, in Spain, the Annual Report of the National Health System 2022 presents a prevalence of ischemic heart disease of 20.9 cases per 1000 inhabitants, more than two-fold in men than in women (29.2 compared to 13.0); while cerebrovascular disease has an incidence of 15.1 cases per 1000 inhabitants, with very close values in men and women (15.9 compared to 14.3). Prevalence of these diseases clearly increases with age, rising after 40 years, and reaching maximum values at 85–94 years [6]; thereby representing an important burden for the national health system.

Populations over 60 years of age are heterogeneous and influenced by several factors, among which we find sex and socioeconomic level [7]; in such a way that over 60 years of age, cardiovascular diseases are more frequent in women and increase with age [8]. Therefore, it is very important to study Cardiovascular Risk Factors (CVRF) separately between women and men [9].

Despite their increase in incidence with advancing age, the World Health Organization (WHO) considers that a large proportion of these diseases could be avoided, and cardiovascular mortality reduced by almost three quarters by adopting changes in lifestyle aimed at controlling CVRF [10].

The objective of this study was to calculate cardiovascular risk in workers of both sexes over 60 years of age and to assess whether there are differences by sex, social class, smoking, and type of job, in order to raise awareness among this population as to what CVRFs are more altered, which is the first step to controlling them. This would be effective in preventing morbidity and mortality in adulthood.

## Materials and methods

### Study design

A cross-sectional study was carried out in 15,057 elderly Spanish workers from different autonomous communities in Spain (the Balearic Islands, Andalusia, the Canary Islands, the Valencian Community, Catalonia, Madrid, Castilla La Mancha, Castile and León, and the Basque Country) and with different labor occupations, the most represented of which are hospitality, construction, commerce, health, public administration, transport, education, industry and cleaning, between January 2019 and June 2020. Workers were selected based on their attendance to periodic occupational medical examinations.

#### Inclusion criteria

- Belonging to one of the participating companies.
- Agreeing to participate in the study and consenting to the use of the data for epidemiological purposes.
- Aged 60 years and over.
- Having the parameters to calculate different cardiovascular risk scales.

**Fig. 1:**
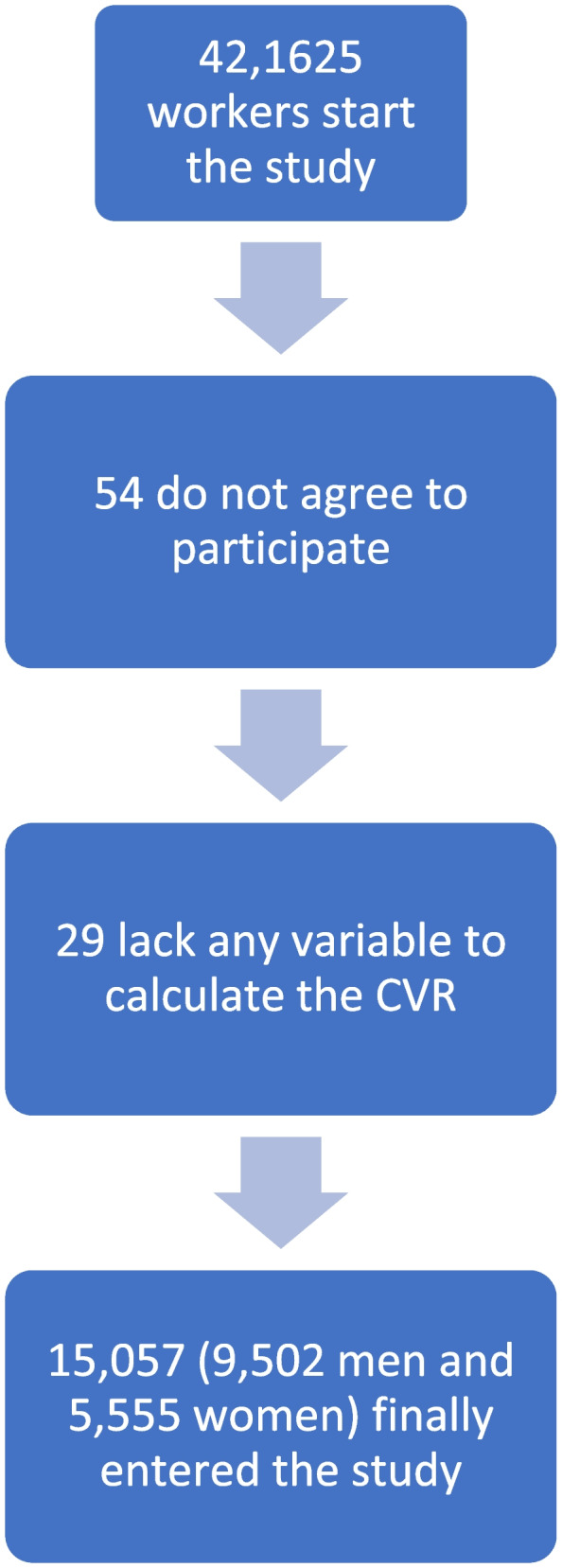
Study participant flow chart

The workers finally included in the study and reasons for exclusion are presented in the flow chart (See Fig. 1).

Anthropometric measurements of height and weight, as well as clinical and analytical data, were collected by health personnel from the occupational health units participating in the study, after standardization of the measurement techniques. According to the International standards for anthropometric assessment of the ISAK [11].

Glycemia, total cholesterol, and triglycerides: These were determined by automated enzymatic methods and HDL by precipitation with dextran sulfate Cl2Mg. LDL was estimated by the *Friedewald* formula (when triglycerides ≤400 mg/dl; LDL = total cholesterol -HDL- triglycerides/5). All results are expressed in mg/dl.

To measure weight (in kilograms) and height (in cm), a height bar scale (model: SECA 700) with an added SECA 220 telescopic height bar was used.

Abdominal waist circumference (WC) was measured in cm using a tape measure: SECA model 20, with an interval of 1–200 cm and millimetric division. For the evaluation, each person was placed in a standing position, feet together and trunk erect, abdomen relaxed and upper limbs hanging down at their sides. The measuring tape was placed parallel to the floor at the level of the last floating rib.

Blood pressure was measured with a well-calibrated OMRON M3 automatic sphygmomanometer after 10 minutes of rest. Three measurements were taken at one-minute intervals, obtaining the mean value of the three.

The following anthropometric indexes and formulas were applied:

- Visceral adiposity index [12] (VAI)
  $${\displaystyle \begin{array}{c} VAI=\left(\frac{{\textrm{WC}}^{\boldsymbol{Male}:}}{39,68+\left(1,88\times \textrm{BMI}\right)}\right)\times \left(\frac{TG}{1,03}\right)\times \left(\frac{1,31}{HDL}\right)\\ {} VAI=\left(\frac{{\textrm{WC}}^{\boldsymbol{Female}:}}{36,58+\left(1,89\times \textrm{BMI}\right)}\right)\times \left(\frac{TG}{0,81}\right)\times \left(\frac{1,52}{HDL}\right)\end{array}}$$
- Dysfunctional adiposity index [13]

[WC/[22.79 + [2.68*BMI]]]*[triglycerides (TG, mmol/L)/1.37]* [1.19/high density lipoprotein-cholesterol (HDL-C, mmol/L)] for males, and [WC/[24.02 + [2.37*BMI]]]* [TG (mmol/L)/1.32]*[1.43/HDL-C (mmol/L)] for females.

- Body shape index (ABSI) [12]
  $$ABSI\equiv \frac{WC}{BMI^{2/3}\times {height}^{1/2}}$$
- Normalized weight-adjusted index (NWAI) [12]

[(weight/10) – (10 x height) + 10] with weight measured in kg and height in m.

- Conicity index [14]
  $$\frac{\textrm{waist}\ \textrm{circumference}\ \left(\textrm{in}\ \textrm{meters}\right)}{0.109}\times 1/\sqrt{\frac{\textrm{weight}\ \left(\textrm{in}\ \textrm{kilogram}\right)}{\textrm{height}\ \left(\textrm{in}\ \textrm{meters}\right)}}$$
- Body Roundness Index [12] (BRI)
  $$\textrm{BRI}=364.2-365.5\times \sqrt{1-\left(\frac{{\left(\textrm{WC}/\left(2\pi \right)\right)}^2}{{\left(0.5\ \textrm{height}\right)}^2}\right)}$$
- Body mass index (BMI) was calculated by dividing weight by height in squared meters [15].
- Waist-to-height ratio was considered risky over 0.50 [16].
- Body surface index [11] (BSA) is calculated from the body surface area (BSA) where w represents weight in kg and h height in cm.
  $$\textrm{BSA}={\textrm{w}}^{0.425}\ast {\textrm{h}}^{0.725}\ast 0.007184\kern0.5em \textrm{BSI}=\frac{\textrm{WEIGHT}}{\sqrt{\textrm{BSA}}}$$
- Relative fat mass [16] Women: 76- (20 x (height/waist))
  Men: 64- (20 x (height/waist)) Height and waist circumference are expressed in meters.
- CUN BAE [15] (Clinic University of Navarra Body Adiposity Estimator):
  $$-44.988+\left(0.503\ \textrm{x}\ \textrm{age}\right)+\left(10.689\ \textrm{x}\ \textrm{sex}\right)+\left(3.172\ \textrm{x}\ \textrm{BMI}\right)-\left(0.026\ \textrm{x}\ {\textrm{BMI}}^2\right)+\left(0.181\ \textrm{x}\ \textrm{BMI}\ \textrm{x}\ \textrm{sex}\right)-\left(0.02\ \textrm{x}\ \textrm{BMI}\ \textrm{x}\ \textrm{age}\right)-\left(0.005\ \textrm{x}\ {\textrm{BMI}}^2\ \textrm{x}\ \textrm{sex}\right)+\left(0.00021\ \textrm{x}\ {\textrm{BMI}}^2\ \textrm{x}\ \textrm{age}\right)$$
- ECORE-BF (Equation Cordoba Estimator Body Fat) [15]

− 97.102 + 0.123 (age) + 11.9 (sex) + 35.959 (LnBMI).

In CUN BAE and ECORE-BF, male is 0 and female 1, and cut-off points for obesity are 35% in women 25% in men.

- Palafolls formula [15]
  $$\textrm{Men}=\left(\textrm{BMI}/\textrm{waist}\right]\ast 10\Big)+\textrm{BMI}.\textrm{Women}=\left(\textrm{BMI}/\textrm{waist}\Big]\ast 10\right)+\textrm{BMI}+10$$
- Deuremberg formula [15]
  $${\displaystyle \begin{array}{cc}1.2\ \textrm{x}\ \left(\textrm{BMI}\right)+0.23\ \textrm{x}\ \left(\textrm{age}\right)-10.8\ \textrm{x}\ \left(\textrm{sex}\right)-5.4& \textrm{Male}=0\kern0.5em \textrm{Female}=1\end{array}}$$

Three atherogenic indexes were calculated [17]:

- Cholesterol/HDL (Castelli index) (considered high when > 5 in men and > 4.5 in women)
- LDL-c/HDL-c (Kannel index) (high values > 3)
- Triglycerides/HDL (high values > 3)

Metabolic syndrome (MetS) was determined using three models:

- NCEP ATP III (National Cholesterol Educational Program Adult Treatment Panel III) [18], which establishes metabolic syndrome when three or more of the following factors are present: waist circumference is greater than 88 cm in women and 102 in men; triglycerides > 150 mg/dl or specific treatment is followed; blood pressure > 130/85 mmHg; HDL < 40 mg/dl in women or < 50 mg/dl in men or specific treatment is followed; and fasting blood glucose > 100 mg/dl or specific glycemic treatment is followed.
- The International Diabetes Federation (IDF) model [19], which considers the presence of central obesity necessary, defined as a waist circumference of > 80 cm in women and > 94 cm in men, in addition to two of the other abovementioned factors for ATP III (triglycerides, HDL, blood pressure, and glycemia).
- The JIS model [20], which follows the same criteria as NCEP ATPIII, but the waist circumference cut-off values are 80 cm in women and 94 cm in men.

In order to determine vascular age, calibrated tables were used. The REGICOR scale, which is an adaptation of the Framingham scale to the characteristics of the Spanish population [21], estimates the risk of suffering a cardiovascular event over a period of 10 years. This scale can be applied to people between 35 and 74 years of age, where the risk is moderate > 5% or high > 10% [22]. The Framingham model^23^ assesses age, sex, HDLc, total cholesterol, systolic blood pressure, hypertension treatment, tobacco use, and diabetes, and can be used in people over 30 years of age. The SCORE scale recommended for Spain estimates the risk of suffering a fatal cerebrovascular event in a period of 10 years [23, 24]. It is applied to people between 40 and 65 years old, and risk is considered moderate at > 4% and high at > 5% 28. The SCORE model [25] considers age, sex, systolic blood pressure, tobacco use, and total cholesterol.

An interesting concept that can be applied to vascular ages using both these models is ALLY (avoidable lost life years), which can be defined as the difference between biological age (BA) and vascular age (VA) [26].

$$\textrm{ALLY}=\textrm{VA}-\textrm{BA}$$

Other indicators related to cardiovascular risk were determined:

- Hypertriglyceridemic waist [27]: waist circumference greater than 94 cm in men and greater than 80 cm in women, and triglycerides greater than 150 mg/dl, or under treatment.
- Waist triglyceride index [28]***:*** Waist circumference (cm) x - triglycerides (mmol).
- Cardiometabolic index [29]

Waist-to-height ratio x atherogenic index triglycerides /HDL-c.

- Triglyceride glucose index [30] = LN (triglycerides [mg/dl] × glycaemia [mg/dl]/2).
- Triglyceride glucose index-BMI, Triglyceride glucose index-waist [31]
  - TyGindex-BMI = TyGindex x BMI
  - TyGindex-waist = TyGindex x waist

Non-alcoholic fatty liver scales:

- Fatty liver index (FLI) [32]
  $$\textrm{FLI}=\left({\textrm{e}}^{0.953}\ast {\log}_{\textrm{e}}\ \left(\textrm{triglycerides}\right)+0.139\ast \textrm{BMI}+0.718\ast {\log}_{\textrm{e}}\ \left(\textrm{ggt}\right)+0.053\ast \textrm{waist}\ \textrm{circumference}-15.745\right)/\left(1+{\textrm{e}}^{0.953}\ast {\log}_{\textrm{e}}\ \left(\textrm{triglycerides}\right)+0.139\ast \textrm{BMI}+0.718\ast {\log}_{\textrm{e}}\ \left(\textrm{ggt}\right)+0.053\ast \textrm{waist}\ \textrm{circumference}-15.745\right)\ \textrm{x}\ 100$$

Cut-off for high risk, 60.

- Hepatic steatosis index (HSI) [33]
  $$\textrm{HSI}=8\ \textrm{x}\ \textrm{ALT}/\textrm{AST}+\textrm{BMI}\ \left(+2\ \textrm{if}\ \textrm{type}\ 2\ \textrm{diabetes}\ \textrm{yes},+2\ \textrm{if}\ \textrm{female}\right)$$
- Zhejian University index (ZJU) [34]
  $$\textrm{BMI}+\textrm{FPG}\ \textrm{mmol}\ \textrm{L}+\textrm{TG}\ \textrm{mmol}\ \textrm{L}+3\ \textrm{ALT}/\textrm{AST}\kern0.5em +2\ \textrm{if}\ \textrm{female}$$
- Fatty liver disease index (FLD) [35]
  $$\textrm{BMI}+\textrm{TG}+3\times \left(\textrm{ALT}/\textrm{AST}\right)+2\times \textrm{Hyperglycemia}\ \left(\textrm{presence}=1;\textrm{absence}=0\right)$$

Values < 28.0 or > 37.0 exclude the possibility of NAFLD

- Lipid accumulation product [36]
  $${\displaystyle \begin{array}{c}\textrm{In}\ \textrm{men}:\left(\textrm{waist}\ \textrm{circumference}\ \left(\textrm{cm}\right)-65\right)\ \textrm{x}\ \left(\textrm{triglyceride}\ \textrm{concentration}\ \left(\textrm{mMol}\right)\right)\\ {}\textrm{In}\ \textrm{women}:\left(\textrm{waist}\ \textrm{circumference}\ \left(\textrm{cm}\right)-58\right)\ \textrm{x}\ \left(\textrm{triglyceride}\ \textrm{concentration}\ \left(\textrm{mMol}\right)\right)\end{array}}$$

Atherogenic dyslipidemia is defined by triglycerides > 150 mg/dL, HDL < 40 mg/dL in men and < 50 mg/dL in women, and normal LDL. If LDL is > 130 mg/dL we speak of lipid triad [37].

##### Fibrosis hepatic scale

- BARD scoring system [38]

The BARD score was calculated by assigning 0 to 2 points to the following parameters: BMI ≥ 28 kg/m2 = 1 point, BMI < 28 kg/m2 = 0 points; AST/ALT ratio ≥ 0.8 = 2 points, AST/ALT ratio < 0.8 = 0 points; diabetes mellitus = 1 point. A total of 2–4 points indicates significant fibrosis.

A smoker was considered to be any person who had regularly consumed at least 1 cigarette/day (or the equivalent in other types of consumption) in the last month, or had quit smoking less than 1 year before.

Social class was determined from the 2011 National Classification of Occupations (CNO-11), based on the proposal of the group of social determinants of the Spanish Society of Epidemiology [39]. It is classified in 3 categories: Class I: Directors/managers, university professionals, athletes, and artists; Class II: Intermediate occupations and self-employed workers without employees; Class III: Unskilled workers.

### Statistical analysis

A descriptive analysis of the categorical variables was carried out, by calculating the frequency and distribution of responses for each one. For quantitative variables, the mean and standard deviation were calculated, whereas for qualitative variables the percentage was calculated. A bivariate association analysis was performed using the χ^2^ test (with a correction using the Fisher’s exact statistical test, when conditions required so) and Student’s *t*-test for independent samples. For the multivariate analysis, binary logistic regression was conducted using the Wald method, with an Odds-ratio calculation and a Hosmer-Lemeshow goodness-of-fit test. Statistical analysis was performed with the SPSS 27.0 program, and a *p* value of < 0.05 was considered statistically significant.

### Ethical considerations and aspects

The study was approved by the Clinical Research Ethics Committee of the Balearic Islands Health Area (n° IB4383/20). All procedures were performed in accordance with the ethical standards of the institutional research committee and with the 2013 Declaration of Helsinki. All patients signed written informed consent documents prior to participation in the study.

## Results

All the anthropometric, sociodemographic, clinical, and analytical values evaluated were more unfavorable in men, except for total cholesterol and LDL-c, and the differences observed between both sexes were statistically significant. Of the people making up the sample, 93% were between 60 and 64 years old, with only the remaining 7% aged between 65 and 69 years. This is logical, since in the case of workers, the retirement age in Spain is 65 years (majority of the sample) although a small proportion of workers can extend their working life to 70 years (forced retirement). About 80% of men and women belonged to social class III, and almost a third of them were smokers, with no significant differences by sex. All data are shown in Table 1.

**Table 1:** Anthropometric, sociodemographic, clinical and analytical values of elderly people

	Women ***n*** = 5555	Men ***n*** = 9502	***p***-value
	Mean (SD)	Mean (SD)	
**Age**	61.8 (1.7)	61.8 (1.8)	0.685
**Height**	157.6 (6.3)	170.3 (6.8)	< 0.0001
**Weight**	67.3 (12.2)	81.7 (13.8)	< 0.0001
**Waist circumference**	74.6 (10.2)	86.2 (10.5)	< 0.0001
**Systolic blood pressure**	131.8 (17.7)	138.5 (17.8)	< 0.0001
**Diastolic blood pressure**	78.1 (10.2)	82.5 (10.3)	< 0.0001
**Total cholesterol**	217.8 (35.6)	204.9 (37.0)	< 0.0001
**HDL-c**	53.4 (8.4)	45.7 (7.9)	< 0.0001
**LDL-c**	142.4 (34.9)	131.7 (35.7)	< 0.0001
**Triglycerides**	109.9 (52.1)	140.8 (76.7)	< 0.0001
**Glycemia**	97.2 (22.1)	106.7 (31.0)	< 0.0001
**ALT**	24.0 (14.3)	29.2 (16.0)	< 0.0001
**AST**	20.0 (6.9)	24.1 (9.9)	< 0.0001
**GGT**	26.5 (24.5)	42.6 (47.2)	< 0.0001
**Creatinine**	0.7 (0.2)	0.9 (0.2)	< 0.0001
	**Percentage**	**Percentage**	***p*-value**
**60–64 years**	93.0	92.9	0.843
**65–69 years**	7.0	7.1	
**Social class I**	4.6	6.2	< 0.0001
**Social class II**	16.3	13.6	
**Social class III**	79.1	80.2	
**Blue-collar**	79.1	80.2	0.105
**White-collar**	20.9	19.8	
**Non-smokers**	69.4	69.4	0.980
**Smokers**	30.6	30.6	

Student’s t-test to assess differences in means, Chi-square t-test to evaluate differences in prevalence, *HDL-c* High density lipoprotein cholesterol, *LDL-c* Low density lipoprotein cholesterol, *ALT* alanine aminotransferase, *AST* Aspartate aminotransferase, *GGT* Gamma Glutamyl Transpeptidase

In the mean values of the different scales differentiated by sex, is worth noting that in the overweight and obesity scales, men obtained worse results than women, except for the formulas in which sex is taken into account. In these cases, the results were significantly more unfavorable for women (CUN-BAE, ECORE-BF, Palafolls, Relative Fat Mass, Deuremberg). The atherogenic indexes analyzed in this study reveal higher mean values in men, with the differences observed between sexes for all scales showing statistical significance. In the non-alcoholic fatty liver risk scales, the results were also more unfavorable for women in the formulas that included sex. In the BARD score system, where sex is not taken into account, the worst results were for men. When applying the ALLY through both the REGICOR scale and the SCORE scale, the potential years of life lost were much higher in men than in women. These results can be seen in Table 2.

**Table 2:** Mean values of different scales related to cardiovascular risk in elderly people by sex

	Women ***n*** = 5555	Men ***n*** = 9502	***p***-value
	Mean (SD)	Mean (SD)	
**Waist to height ratio**	0.47 (0.06)	0.51 (0.06)	< 0.0001
**Body mass index**	27.1 (4.7)	28.1 (4.2)	< 0.0001
**CUN BAE**	40.5 (5.1)	30.0 (4.9)	< 0.0001
**ECORE-BF**	40.5 (6.1)	30.1 (5.3)	< 0.0001
**Relative fat mass**	33.0 (5.5)	24.0 (4.7)	< 0.0001
**Palafolls formula**	40.7 (5.0)	31.4 (4.4)	< 0.0001
**Deuremberg formula**	41.3 (5.7)	31.8 (5.1)	< 0.0001
**Body surface index**	51.7 (7.1)	58.6 (7.4)	< 0.0001
**Body roundness index**	3.0 (1.2)	3.5 (1.2)	< 0.0001
**Body shape index**	0.066 (0.006)	0.072 (0.006)	< 0.0001
**Visceral adiposity index**	3.4 (2.0)	9.1 (6.1)	< 0.0001
**Dysfunctional adiposity index**	0.9 (0.5)	1.1 (0.7)	< 0.0001
**Conicity index**	1.0 (0.1)	1.1 (0.1)	< 0.0001
**Normalized weight-adjusted index**	1.1 (1.2)	1.2 (1.2)	< 0.0001
**Fatty liver index**	25.0 (22.9)	45.9 (26.4)	< 0.0001
**Hepatic steatosis index**	38.6 (6.6)	37.7 (6.1)	0.001
**Zhejiang University index**	39.4 (5.9)	38.8 (5.4)	0,008
**Fatty liver disease index**	32.1 (5.5)	33.2 (4.9)	< 0.0001
**Lipid accumulation product**	21.9 (20.5)	35.5 (29.7)	< 0.0001
**Bard scoring system**	1.7 (0.8)	2.1 (0.9)	< 0.0001
**Triglyceride glucose index**	8.5 (0.5)	8.8 (0.6)	< 0.0001
**Triglyceride glucose index-BMI**	230.4 (47.0)	247.7 (44.7)	< 0.0001
**Triglyceride glucose index-waist**	633.5 (103.7)	758.2 (115.2)	< 0.0001
**Triglyceride glucose index-WtHR**	4.0 (0.7)	4.5 (0.7)	< 0.0001
**Waist triglyceride index**	94.0 (50.8)	138.9 (81.2)	< 0.0001
**Cardiometabolic index**	1.0 (0.7)	1.7 (1.1)	< 0.0001
**ALLY vascular age SCORE**	8.9 (5.3)	13.1 (7.6)	< 0.0001
**SCORE scale**	2.4 (2.0)	5.7 (3.7)	< 0.0001
**ALLY vascular age Framingham**	12.1 (13.6)	15.7 (11.1)	< 0.0001
**REGICOR scale**	3.9 (2.4)	4.4 (3.0)	< 0.0001
**Cardiovascular disease risk**	12.3 (7.1)	26.2 (8.8)	< 0.0001
**Framingham categories**	10.1 (4.7)	19.1 (9.2)	< 0.0001
**n° factors of metabolic syndrome NCEP ATPIII**	1.9 (1.2)	2.3 (1.2)	< 0.0001
**n° factors of metabolic syndrome JIS**	2.0 (1.3)	2.8 (1.2)	< 0.0001
**Atherogenic index total cholesterol/HDL-c**	4.2 (0.9)	4.6 (1.2)	< 0.0001
**Atherogenic index triglycerides/HDL-c**	2.1 (1.2)	3.2 (2.0)	< 0.0001
**Atherogenic index LDL-c/HDL-c**	2.7 (0.8)	3.0 (1.0)	< 0.0001

*CUN BAE* Clínica Universitaria Navarra Body Adiposity Estimator, *ECORE BF* Equation Cordoba Estimator body fat, *BMI* Body mass index, *WtHR* Waist to height ratio, *ALLY* Avoidable lost life years, *REGICOR* Registre Gironi del Cor, *SCORE* Systematic Coronary Risk Evaluation, *LDL-c* Low density Lipoprotein cholesterol, *MS NCEP ATPIII* Metabolic syndrome National Cholesterol Education Program Adult Treatment Panel III, *MS JIS* Metabolic syndrome Joint Interim Statement, *AI* Atherogenic index, *HDL-c* High density Lipoprotein cholesterol, Student’s t-test to assess differences in means

Prevalence of altered values in the overweight and obesity scales presents a higher percentage for men in all the formulas used, and in all of them significantly. Only by applying the CUN-BAE formula did we find a slightly higher result in women, but without statistical significance, therefore it should be rejected.

The percentage of the population with hypertension was also significantly higher in men than in women. As far as laboratory values are concerned, we found that Triglyceride and Glucose figures were more frequent in men, while women had much higher total Cholesterol and LDL-c figures much more frequently.

When evaluating MetS using the three formulas, the percentage of men who present MetS by both the NCEP ATPIII and JIS criteria was much higher in men, with significant statistical significance. However, when evaluating it according to the IDF criteria, this percentage was higher in women, despite it lacking a significant value.

Atherogenic indexes also showed higher percentages in men, all with statistical significance.

As for the risk scales for non-alcoholic fatty liver disease, the results show a higher risk of non-alcoholic fatty liver disease in men regardless of whether the sex factor is included in the formula. In most cases, as can be seen in Table 3, the differences observed show statistical significance.

**Table 3:** Prevalence of altered values of different scales related to cardiovascular risk in elderly people by sex

	Women ***n*** = 5555	Men ***n*** = 9502	***p***-value
	Percentage	Percentage	
**Waist to height ratio > 0.50**	28.4	51.3	< 0.0001
**Body mass index obesity**	23.3	29.4	< 0.0001
**CUN BAE obesity**	85.8	85.4	0.687
**ECORE-BF obesity**	81.5	84.2	< 0.0001
**Relative fat mass obesity**	58.1	43.8	< 0.0001
**Palafolls formula obesity**	89.1	94.6	< 0.0001
**Deuremberg formula obesity**	93.2	99.5	< 0.0001
**Hypertension**	42.7	57.9	< 0.0001
**Total cholesterol ≥ 200 mg/dl**	68.6	53.7	< 0.0001
**LDL-c ≥ 130 mg/dl**	62.4	51.1	< 0.0001
**Triglycerides ≥ 150 mg/dl**	16.7	32.7	< 0.0001
**Glycemia 100–125 mg/dl**	25.7	35.7	< 0.0001
**Glycemia ≥ 126 mg/dl**	5.6	13.2	< 0.0001
**Metabolic syndrome NCEP ATPIII criteria**	30.8	42.7	< 0.0001
**Metabolic syndrome IDF criteria**	21.2	20.7	0.455
**Metabolic syndrome JIS criteria**	34.4	58.6	< 0.0001
**Atherogenic dyslipidemia**	10.6	15.5	< 0.0001
**Lipid triad**	3.1	4.4	< 0.0001
**Hipertriglyceridemic waist**	2.6	11.0	< 0.0001
**Atherogenic index total cholesterol/HDL-c moderate-high**	31.6	33.5	< 0.0001
**Atherogenic index triglycerides/HDL-c high**	16.2	42.5	< 0.0001
**Atherogenic index LDL-c/HDL-c high**	34.0	46.2	< 0.0001
**SCORE scale high**	15.7	62.4	< 0.0001
**REGICOR scale high-very high**	2.9	5.4	< 0.0001
**Fatty liver index high risk**	10.7	32.7	< 0.0001
**Hepatic steatosis index**	59.3	63.1	0.008
**Zhejiang University index**	52.4	54.4	0.501
**Fatty liver disease index**	60.6	67.6	0.002

*CUN BAE* Clínica Universitaria Navarra Body Adiposity Estimator, *ECORE BF* Equation Cordoba Estimator body fat, *LDL-c* Low density Lipoprotein cholesterol, *NCEP ATPIII* National Cholesterol Education Program Adult Treatment Panel III, *IDF* International Diabetes Federation, *JIS* Joint Interim Statement, *HDL-c* High density Lipoprotein cholesterol, *REGICOR* Registre Gironi del Cor, *SCORE* Systematic Coronary Risk Evaluation, Chi-square t-test to evaluate differences in prevalence

In the multivariate analysis, when making the comparison between men and women, all the parameters measured were more unfavorable in men except for Relative fat mass obesity, Total Cholesterol, and LDL-c.

In the analysis of social class, social class III was compared to social class II plus social class I. In this case, only four variables showed significant differences: diabetes, metabolic syndrome according to the ATP-III criteria, and metabolic syndrome according to the JIS criteria were more frequent in social classes I and II; while high fatty liver disease index occurred more frequently in social class III compared to the other two.

Blue-collar workers performed worse than white-collar workers in all variables that increase cardiovascular risk with statistical significance (obesity, diabetes, arterial hypertension, metabolic syndrome, and AI LDL-c/HDL-c high). They also presented a higher cardiovascular risk with both the REGICOR and SCORE scales, as well as a worse result in the Bard scoring system high. On the other hand, white-collar workers only presented worse results in the Lipid accumulation product high.

Smoking only showed significant results in the calculation formulas for obesity, total cholesterol, LDL-c and cardiovascular risk. Except for cardiovascular risk, in the rest of the cases it presented an OR less than 0, which must be interpreted as a protective factor. The complete data can be consulted in Table 4.

**Table 4:** Multivariate analysis using binary logistic regression

	Male	Social class III	Blue collar	Smokers
	OR (95% CI) ***p***-value	OR (95% CI) ***p***-value	OR (95% CI) ***p***-value	OR (95% CI) ***p***-value
**WtHR > 0.50**	2.65 (2.47–2.84) < 0.0001	1.01 (0.86–1.20) 0.867	1.14 (1.04–1.26) 0.007	0.95 (0.89–1.02) 0.185
**Obesity BMI**	1.37 (1.27–1.48) < 0.0001	1.05 (0.88–1.27) 0.582	1.18 (1.06–1.31) 0.003	0.92 (0.85–0.99) 0.049
**Obesity CUN BAE**	0.97 (0.89–1.07) 0.585	1.11 (0.89–1.38) 0.359	1.06 (0.93–1.20) 0.394	0.87 (0.79–0.95) 0.003
**Obesity ECORE-BF**	1.21 (1.11–1.32) < 0.0001	1.07 (0.87–1.32) 0.522	1.07 (0.95–1.21) 0.242	0.87 (0.79–0.95) 0.003
**Obesity RFM**	0.56 (0.52–0.60) < 0.0001	0.97 (0.82–1.14) 0.676	1.20 (1.10–1.32) < 0.0001	0.93 (0.87–0.99) 0.048
**Obesity Palafolls**	2.15 (1.90–2.43) < 0.0001	1.06 (0.79–1.43) 0.684	1.10 (0.93–1.30) 0.278	0.86 (0.76–0.98) 0.026
**Diabesity**	1.86 (1.65–2.10) < 0.0001	0.83 (0.62–1.11) 0.204	1.69 (1.42–2.02) < 0.0001	0.98 (0.87–1.10) 0.712
**Hypertension**	1.84 (1.72–1.97) < 0.0001	1.09 (0.92–1.28) 0.316	1.21 (1.11–1.33) < 0.0001	0.99 (0.93–1.07) 0.838
**Total cholesterol high**	0.53 (0.50–0.57) < 0.0001	0.94 (0.80–1.10) 0.431	0.96 (0.87–1.05) 0.335	0.92 (0.86–0.99) 0.019
**LDL-c high**	0.63 (0.59–0.67) < 0.0001	0.95 (0.81–1.11) 0.523	1.04 (0.95–1.14) 0.370	0.93 (0.87–0.99) 0.045
**Triglycerides high**	2.42 (2.23–2.63) < 0.0001	0.94 (0.79–1.13) 0.519	0.95 (0.86–1.06) 0.350	0.97 (0.90–1.05) 0.469
**Diabetes**	1.97 (1.82–2.14) < 0.0001	0.74 (0.61–0.90) 0.002	1.78 (1.58–2.00) < 0.0001	1.03 (0.95–1.12) 0.446
**MS NCEP-ATPIII criteria**	1.67 (1.55–1.79) < 0.0001	0.84 (0.71–0.99) 0.040	1.26 (1.14–1.39) < 0.0001	0.99 (0.93–1.07) 0.906
**MS IDF criteria**	0.97 (0.89–1.05) 0.429	0.92 (0.76–1.11) 0.374	1.02 (0.91–1.15) 0.690	1.01 (0.93–1.10) 0.808
**MS JIS criteria**	2.69 (2.51–2.88) < 0.0001	0.84 (0.71–0.99) 0.035	1.30 (1.18–1.43) < 0.0001	0.97 (0.91–1.05) 0.468
**Atherogenic dyslipidemia**	1.55 (1.40–1.72) < 0.0001	0.96 (0.76–1.20) 0.703	0.98 (0.86–1.12) 0.730	1.01 (0.91–1.11) 0.925
**Lipid triad**	1.41 (1.18–1.69) < 0.0001	0.87 (0.58–1.31) 0.498	1.12 (0.88–1.43) 0.361	0.96 (0.81–1.16) 0.692
**Hypertriglyceridemic waist**	1.41 (1.18–1.69) < 0.0001	0.87 (0.58–1.31) 0.498	1.12 (0.88–1.43) 0.361	0.96 (0.81–1.16) 0.692
**AI CT/HDL-c moderate-high**	1.09 (1.01–1.17) 0.023	0.93 (0.78–1.10) 0.382	1.18 (1.07–1.31) 0.001	0.93 (0.86–0.99) 0.048
**AI triglycerides/HDL-c high**	3.83 (3.53–4.16) < 0.0001	1.02 (0.86–1.21) 0.834	0.95 (0.86–1.05) 0.276	0.96 (0.87–1.05) 0.264
**AI LDL-c/HDL-c high**	1.66 (1.55–1.78) < 0.0001	0.97 (0.82–1.14) 0.695	1.22 (1.11–1.34) < 0.0001	0.97 (0.90–1.04) 0.335
**REGICOR scale high-very high**	1.97 (1.64–2.36) < 0.0001	0.90 (0.61–1.33) 0.595	1.28 (1.00–1.63) 0.049	3.15 (2.69–3.69) < 0.0001
**SCORE scale high-very high**	22.51 (19.87–25.49) < 0.0001	0.98 (0.80–1.21) 0.873	1.18 (1.05–1.32) 0.007	17.88 (15.80–20.24) < 0.0001
**Fatty liver index high risk high**	4.06 (3.66–4.50) < 0.0001	0.84 (0.68–1.04) 0.107	1.06 (0.93–1.20) 0.380	0.95 (0.87–1.04) 0.227
**Fatty liver disease index high**	1.36 (1.15–1.62 < 0.0001	1.44 (1.00–2.07) 0.048	0.82 (0.64–1.04) 0.106	1.04 (0.87–1.24) 0.673
**Bard scoring system high**	2.10 (1.95–2.26) < 0.0001	0.92 (0.77–1.11) 0.397	1.24 (1.12–1.38) < 0.0001	1.00 (0.93–1.09) 0.925
**Lipid accumulation product high**	1.69 (1.58–1.81) < 0.0001	1.05 (0.89–1.23) 0.561	0.84 (0.76–0.92) < 0.0001	0.94 (0.87–1.01) 0.076

*WtHR* Waist to heigh ratio, *BMI* Body mass index, *CUN BAE* Clínica Universitaria Navarra Body Adiposity Estimator, *ECORE BF* Equation Cordoba Estimator body fat, *RFM* Relative Fat Mass, *LDL-c* Low density Lipoprotein cholesterol, *MS NCEP ATPIII* Metabolic syndrome National Cholesterol Education Program Adult Treatment Panel III, *MS IDF* Metabolic syndrome International Diabetes Federation, *MS JIS* Metabolic syndrome Joint Interim Statement, *AI* Atherogenic index, *CT/HDL-c* Atherogenic index Total Cholesterol/High density Lipoprotein cholesterol, *REGICOR* Registre Gironi del Cor, *SCORE* Systematic Coronary Risk Evaluation

## Discussion

We studied a sample of 15,057 workers of both sexes between 60 and 69 years of age. Of these, 5555 were women and 9502 men, corresponding to different social classes, and with 80% blue-collar and 20% white-collar workers.

When comparing the calculation of obesity according to BMI with the rest of the formulas used, we observe that the percentage of obese in both sexes was more than double that of any of the other formulas. Various studies have already warned of these differences, obtaining results of two to six times higher by methods other than BMI, which implies that BMI has a good specificity for identifying excess weight, but low sensitivity [40, 41].

When studying the mean values of the different scales with which we measured overweight and obesity, we found more unfavorable results for men except in the formulas that include sex in their calculation. In these cases, the results were significantly more unfavorable for women (CUN-BAE, ECORE-BF, Palafolls, Relative Fat Mass, Deuremberg), which coincides with the results obtained in other studies where the sex factor seems to bear an influence [42, 43].

The prevalence of MetS defined by the three criteria used (NCEP ATPIII, IDF, JIS) was high in both men and women; with a significantly higher percentage in males, except for the IDF, which did not present significant differences between sexes. These data differ from those obtained by other authors, in which MetS is more common in women over 60 years of age [44–46]. Continuing the comparison with the study by Shasha et al., our results do not coincide either with regard to triglyceride or glycemia values, which we found to be significantly higher in men, while they obtained the highest triglycerides in women. Neither did they find any differences between sexes in blood glucose levels. However, there was a coincidence in figures for higher total cholesterol and LDL-c in women than in men, with a significantly high value in both studies.

It is possible that these results are due to differences in the sample, since in our study the oldest patients were 69 years old, whereas in the aforementioned study the sample evaluated the population up to 80 years of age. The authors refer in their article to the fact that older women were significantly more likely to have MetS, abdominal obesity, hypertriglyceridemia, and lower HDL-c levels, which would favor this hypothesis.

Non-alcoholic fatty liver disease (NAFLD) is a chronic, progressive disease that can be asymptomatic and increases cardiovascular risk factors [47]. Much of the published literature finds a higher prevalence of NAFLD in men [48, 49]. However, we know that as age increases, so does the prevalence of NAFLD, and it tends to be more common in women [50, 51]. For this reason, we considered it interesting to assess whether a similar alteration occurred in our population. In our study, the NAFLD indexes give us a significantly higher percentage in men in all cases except for the Zhejiang University index. Nonetheless, even with a higher percentage in men than in women, it does not present statistical significance. Thus, we do not observe this reversal by sex in NAFLD above 60 years of age.

When we calculated the potential years of life lost (ALLY) using both the REGICOR scale [21] and the SCORE scale [52], these were much higher in men than in women. This result is reasonable, since sex is involved in both formulas. The main variable constitutes a value that increases cardiovascular risk. Having obtained more unfavorable values for men in the other variables that are part of the formula, and as the average age and percentage of smokers is the same in both sexes, the potential years of life lost must necessarily offer a higher result in men, and present much higher cardiovascular risk (CVR) with statistical significance.

In the multivariate analysis using binary logistic regression, being a white-collar/blue-collar worker, being male, tobacco use, and social class III were established as covariates.

When comparing men versus women, the multivariate analysis found an OR greater than 1 in all the formulas studied except for Relative fat mass obesity, Total Cholesterol, and LDL-c, with an OR less than 1. What this tells us is that all the factors studied occur more frequently in males except for the three factors mentioned with an OR less than 1. Levels of elevated cholesterol are known to be lower in younger women compared to men, while they increase after menopause [53], which is in agreement with our results when treating our entire sample of women aged 60 years or over. Atherogenic indexes also showed higher percentages in men, all with statistical significance. This alerts us to a higher risk of suffering a cardiovascular event.

Although at present we must consider whether high cholesterol figures in the population over 60 years decrease mortality from all causes and increase survival, Liang et al. found an association between higher cholesterol levels and a decreased risk of all-cause and non-cardiovascular mortality in the elderly [54]. Hence, more studies are needed in order to clarify the relationship between high cholesterol levels and longer survival in the elderly.

When analyzing social classes, we found a higher risk of obesity, diabetes, hypertension, MetS, atherogenic index, CVR, and BARD scoring system in blue-collar compared to white-collar workers. These results are consistent with other published studies that found that shift work is associated with an increased risk of CVD [55]. Socioeconomic and demographic differences are associated with an uneven distribution of health [56], in such a way that socioeconomic level presents an inverse association with the prevalence of MetS and diabetes mellitus, with a worse health status in the population with less purchasing power [57].

Blue-collar workers with low wages and limited education are more likely to develop unhealthy lifestyles and have poorer living conditions than white-collar workers. A study by Prihartono et al. in 2018 detected differences in CVRF prevalence between white-collar and blue-collar workers by obtaining diagnostic information from the health care provider, in such a way that, according to these reports, the prevalence of CVRF was higher in white-collar than in blue-collar workers. However, when CVRFs were assessed by symptoms, the prevalence of CVRFs was higher in blue-collar workers. This difference may be due to socioeconomic class, which would determine access to health services [58]. The latter is very important in order to detect CVRFs in blue-collar workers, reduce avoidable lost life years, and increase quality of life.

It is noteworthy that only in diabetes, metabolic syndrome, and Fatty liver disease index high do the results obtained coincide (with statistical significance) between the social classes with lower purchasing power and being a blue-collar worker, which could be the seed of future research.

Regarding the smoking habit, we found that in all the formulas to evaluate obesity, the OR was less than 1 with very narrow confidence intervals. This indicates that smoking is a protective factor against obesity, meaning that there is a strong relationship between smoking and not being obese. This may be due to lower food intake and increased metabolism produced by nicotine and consequently a lower weight in smokers, an effect already known and that in no case would justify recommending smoking to avoid these pathologies [59].

Likewise, when calculating CVR (REGICOR) in smokers versus non-smokers, we found an OR of 3.15. This indicates an almost 76% higher probability of suffering a cardiovascular event among smokers, thereby consolidating the need to give up smoking. This association between smoking and CVR is consistent with that published in other studies, with a strong association between smoking and CVR [60, 61].

### Strengths and limitations

Some limitations of the study should also be considered.

Our study has a cross-sectional design, which does not allow causal relationships to be established, so no conclusions can be drawn about changes in anthropometric measurements over time. Second, the population of this study was ethnically homogeneous, as all the patients in this study were Spanish, which could limit the generalization of our findings. Third, since the participants are workers attending their annual medical check-up at the prevention service, a selection bias may occur, since only those who are more concerned about their health or who get sick less often attend.

## Conclusions

Anthropometric, sociodemographic, clinical and analytical values are more unfavorable in men, except Total cholesterol and LDL-c. CVR and risk of developing NAFLD is also higher in men.

Blue-collar workers have worse health outcomes (obesity, diabetes, hypertension, MetS, Atherogenic index) and higher CVR than white-collar workers, which should make us alert in order to act to prevent these factors, reduce morbidity and mortality among these people, and increase their quality of life.

Smokers over 60 years of age have a much higher CVR than non-smokers. Due to population aging, it would be interesting to have CVR calculation tables for people over 70 years of age, which could give rise to new investigations.

## Data Availability

Data available on request due to restrictions (privacy, ethical). Contact corresponding author.
